# Relative Validity and Reproducibility of a Quantitative Food Frequency Questionnaire for Adolescents with Type 1 Diabetes: Validity of a Food Frequency Questionnaire

**DOI:** 10.1155/2014/976508

**Published:** 2014-08-27

**Authors:** Rosana de Moraes Borges Marques, Amanda Cristine de Oliveira, Sheylle Almeida da Silva Teles, Maria Luiza Ferreira Stringuini, Nélida Shimid Fornés, Giulliano Gardenghi

**Affiliations:** ^1^Faculdade de Nutrição, Universidade Federal de Goiás, Rua 227, Quadra 68 s/n, Setor Leste Universitário, 74605-080 Goiânia, GO, Brazil; ^2^Hospital ENCORE, Rua Gurupi, Quadra 25 lt-6, Vila Brasília, 74905-350 Aparecida de Goiânia, GO, Brazil; ^3^Centro de Estudos Avançados e Formação Integrada, Rua T-28, No. 1806, Setor Bueno, 74215-040 Goiânia, GO, Brazil; ^4^Hospital e Maternidade São Cristóvão, Rua Américo Ventura, No. 123, Mooca, 03128-020 São Paulo, SP, Brazil

## Abstract

*Background.* Food frequency questionnaires are used to assess dietary intake in epidemiological studies.* Objective.* The aim of the study was to assess the relative validity and reproducibility of a quantitative food frequency questionnaire (QFFQ) for adolescents with type 1 diabetes. Methods: Validity was evaluated by comparing the data generated by QFFQs to those of 24-hour recalls (24 hrs). QFFQs were applied twice per patient to assess reproducibility. Statistical analysis included performing *t*-tests, obtaining Pearson correlation coefficients when necessary, correcting measurements for randomness by the weighted* kappa* method, calculating intraclass correlation coefficients, and generating Bland-Altman plots (*P* < 0,05).* Results.* The total energy and nutrient intake as estimated by the QFFQs were significantly higher than those from 24 hrs. Pearson correlation coefficients for energy-adjusted, deattenuated data ranged from 0.32 (protein) to 0.75 (lipid, unsaturated fat and calcium). Weighted* kappa* values ranged from 0.15 (vitamin C) to 0.45 (calcium). Bland-Altman plots indicated acceptable validity. As for reproducibility, intraclass correlation coefficients ranged from 0.24 (calcium) to 0.65 (lipid), and the Bland-Altman plots showed good agreement between the two questionnaires. Conclusion: The QFFQ presented an acceptable ability to classify correctly and with good reproducibility, adolescents with type 1 diabetes according to their levels of dietary intake.

## 1. Introduction

The goals of type 1 diabetes treatment in adolescents are to keep patients free of symptoms, prevent acute and chronic complications of hyperglycaemia, avert episodes of hypoglycaemia, control weight, prevent dyslipidemia, and maintain normal growth and development rates. To ensure the success of this approach, it is essential to know their dietary habits in order to modify them, as well as for continued evaluation and follow-up of patients. Despite its importance, few studies about food consumption and its relation with the control and management of this disease are found in the literature [1].

Several methods have been developed to assess dietary intake of individuals and populations. Since each method has its advantages and limitations, choice must be guided by its adequacy to the target population and the goals of the study. Furthermore, for the reliability of the analysis the method must be highly reproducible and also must have been tested in a posterior validation process [2].

The use of food frequency questionnaires is a low cost method to assess dietary intake and is often used in epidemiological studies, since it allows correlating diet and the occurrence of nontransmissible chronic illnesses. The aim of this method is to evaluate the frequency of intake of certain foods, or food groups, over a specific period of time [3–5].

Considering factors as the prevalence of diabetes among adolescents, its substantial correlation with dietary intake and impact on growth and development, including also the lack of specific assessment tools for this population, the present study evaluated the reproducibility and relative validity of a quantitative food frequency questionnaire for type 1 diabetes adolescent patients.

## 2. Materials and Methods

### 2.1. Development of the QFFQ

The participants in this study were DM1 adolescents of both sexes who were followed as patients at the Endocrinology Outpatient Clinic of the Clinical Hospital (CH) of the Goiás Federal University (EOC-CH). All were volunteers whose consent was requested, in the presence of their legal guardians, about their interest in taking part of the research and, upon being informed about the study, they signed the Free and Informed Consent Formulary (FICF) in accordance with the guidelines of the Medical Research Ethics Committee of Goiás Federal University (GFU).

At the time the study was outlined, there were 170 adolescents registered at the EOC-CH, 20% of whom (34 adolescents) were selected to evaluate the food items that were to be included in the quantitative food frequency questionnaire (QFFQ).

In order to identify the food items consumed by the study cohort, an adult QFFQ was used (validated previously) [6]. This questionnaire covered the previous three months of food consumption, using open questions that allowed the inclusion of new food items, portion sizes, and usual preparations. Each patient also filled two 24-hour recalls (24hRs), one on the day of the interview and another 15–20 days later. The interviewers were undergraduate students of Nutrition at the GFU who had been previously trained. Interviews were carried out under supervision of the researchers.

Nutrient content calculations were performed on the data from dietary surveys based on the Brazilian reference tables for the chemical composition of food items [7, 8]. Lists were compiled with the percentage contribution (PC) of all food items towards each nutrient, in accordance with the statistical analysis technique of weighted proportions according to Block et al. [9], Haile et al. [10], Willett [11], and Flegal et al. [12]. To this end, the following formula was used: PC (%) = 100 × Σ(specific nutrient content per food item)/Σ(nutrient content in all food items).

All food items were classified by PC value, and those with a PC equal to or lower than 85% were included into the questionnaire. The preliminary list resulting from this selection included few dietetic and low calorie food items, due to the low frequency of consumption by the study group.

To identify the consumption frequency (cf) for each food item that was in the QFFQ, we defined nine frequency unit categories of classification. This assessment was quantified by attributing weights (*Sn*, where *n* is the category number) to each category (cf) based on the frequency in the previous three months [13].

The mean value of *S*6 = 1 was defined for items ingested daily. Weights for the other categories were obtained according to the following formula for a given food item ingested between *a* and *b* times in the past three months [13]: *Sn* = (1/90)×(*a* + *b*)/2. Consumption frequency categories (f) and their respective weights are as follows:

(f1) never or less than once a month; *S*1 = 0;
(f2) once a month; *S*2 = 0,016[6/2 = 3∗0.0055 = 0.016];
(f3) twice to four times a month; *S*3 = 0,099[12 + 24/2 = 18∗0.0055 = 0.09];
(f4) twice to four times a week; *S*4 = 0,43[52 + 104/2 = 78∗0.0055 = 0.43];
(f5) five or six times a week; *S*5 = 0,79[130 + 156/2 = 143∗0.0055 = 0.79];
(f6) once a day; *S*6 = 1;
(f7) twice or thrice a day; *S*7 = 2,5[360 + 540/2 = 450∗0.0055 = 2.5];
(f8) four or five times a day; *S*8 = 4,5[720 + 900/2 = 810∗0.0055 = 4.5];
(f9) six times a day; *S*9 = 6[1080∗0.0055 = 5.9].

The usual portion sizes were defined from the reported portions in the two 24hRs. These were classified relative to the 25th, 50th, and 75th percentiles, which marked the thresholds for small, medium, and large portions, respectively.

The final format of the QFFQ, corresponding to the three preceding months, comprised 106 food items divided into eleven groups, namely, dairy products, legumes, meat and eggs (with or without apparent fat), cereals and derivative products, pasta and snacks, sugar and sweets, fruits, green leaves, fats, spices and seasonings, and nonalcoholic beverages. The reported dietary intake for a given product, thus, is quantified by multiplying the quantities in grammas or milliliters by the aforementioned weights (*Sn*), according to the consumption frequency category.

### 2.2. Relative Validity and Reproducibility

**Figure 1:**
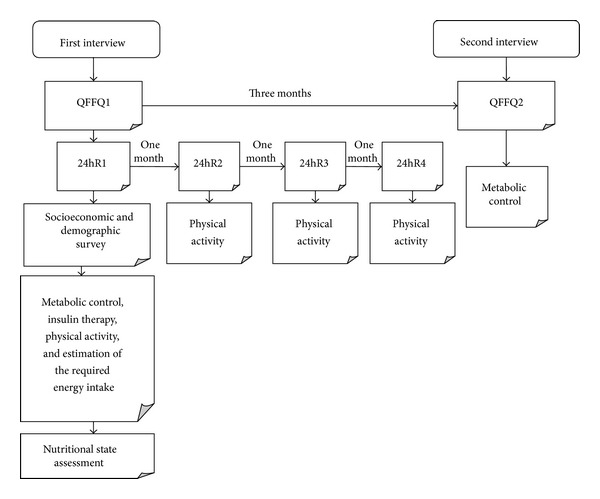
Protocol for validation and reproducibility analysis of the proposed QFFQ for type 1 diabetes adolescent.

Relative validity was assessed by comparing QFFQ results to those of a reference method, the 24-hour recall (24hR). Reproducibility was evaluated by comparing results of two separate applications of the same questionnaire. The overall design of the study is outlined in Figure 1.

Participants were adolescents of both sexes who were regular patients at the Endocrinology Outpatient Clinics of the GGH and EOC-CH, both in the city of Goiânia, Brazil. Inclusion criteria were of ages between ten and 18 years and a positive DM1 diagnosis; patients were not included if they had other types of diabetes, celiac disease, growth hormone deficiency, or chromosomal abnormalities. Girls who were pregnant at the time of selection or became pregnant at any time during the course of the study were also not included.

We recruited 84 patients. Data were collected from April 2008 to July 2009. A guideline basis was constructed in order to standardize the procedures of data collection.

The 24hR, a validated method [14] used in national [2, 15] and international [14, 16] validation studies, allowed us to register meal times and types, as well as the food items, their preparations, and quantities. The customary measures reported were converted to grammas or milliliters. Nutritional content was calculated by reference to the national tables of chemical composition of food items [7, 8] and to package labels.

The following dietary variables were analyzed: total energetic content, carbohydrates, protein, lipids, saturated fats, unsaturated fats, total cholesterol, dietary fiber, vitamin C, calcium, iron, and zinc.

Variables that did not follow a normal distribution underwent Neperian logarithmic transformation to generate an approximate Gaussian distribution, which was successfully accomplished for all of them. Mean and standard deviation values for total energy, macronutrients, and micronutrients were determined for both QFFQ and the four 24hRs. The differences between mean values obtained by each survey technique were calculated by Student's *t*-test.

Validity was assessed by using the Pearson correlation coefficient for crude, deattenuateddata, and again after they were adjusted for energy content. Deattenuation, which corrects intrapersonal variability, was carried out according to the procedure described by Beaton and colleagues [17]: *r*_*d*_ = *r*_*p*_ × (1 + *λ*_*x*_/*n*_*x*_)^1/2^, where *r*_*d*_ is the deattenuated coefficient, *r*_*p*_ is the observed coefficient, *λ*_*x*_ is the ratio of intrapersonal variation, and *n*_*x*_ is the number of surveys per subject. Values were adjusted for energy by means of regression using the residue method, whereby the total energy content was considered an independent variable and the nutrient itself was considered a dependent one [11].

The nutrients under study were categorized in quartiles of intake in order to correlate the mean values of the QFFQ and those of the 24hR. Concordance and extreme discordance between methods were estimated by the percentage of patients classified into the same quartile, in opposed quartiles and in adjoining quartiles. Reliability of analyses was assessed by using the weighed* kappa* method, which corrects concordance measurements for chance events; results were considered acceptable when more than 50% of individuals were correctly classified, less than 10% fell on opposite quartiles, and the weighed* kappa* was more than 0.4, so that the possibility of false-negative associations between diet and illness in epidemiological studies would be kept to a minimum.

The intraclass correlation coefficient (ICC) was used to assess reproducibility of the QFFQ in two different forms, that is, for energy-adjusted and unadjusted values. To assess the agreement between both QFFQ, differences were compared to mean values and a plot comparing the two measurements was drawn as suggested by Bland and Altman [18].

*P* values lower than 0.05 were considered significant and correlations were found to be moderate between 0.4 and 0.7. Statistical analyses were conducted using two different programs:* Statistical Analysis System* (SAS), version 9.2, and* Statistical Package of Social Sciences* (SPSS), version 18.0.

The study was approved by the Ethics Committee in Human and Animal Medical Research of both CH (protocol 042/07) and GGH (protocol 383/08). All patients and legal guardians were informed about the goals and procedures of the study and, upon consent on volunteer participation, signed the FICF.

## 3. Results

At the end of the data collection period, from the 84 patients that were initially included in the study group, 14 (17.0%) returned incomplete dietary reports, leaving 70 adolescents (58.6% females) for the study to be carried out on. The mean age was 14 years (SD = ±2.5 years), the mean monthly* per capita* income was 99.68 USD (ranging from 28.48 to 360.28 USD), and the mean school attendance was seven years (7–16).

Table 1 presents the means for the estimated total energy and nutrient intake for QFFQs and 24hRs. It is of note that QFFQ overestimated intake relative to 24hR. This trend was statistically significant for all analysed measurements. The QFFQ : 24hR ratio of individual means varied from 1.14 (total cholesterol and zinc) to 2.95 (vitamin C).

As for Pearson correlation analyses, the estimates for total energetic intake, carbohydrate, lipids, saturated fat and unsaturated fats, dietary fibre, calcium, iron, and zinc all fell in the moderate range (values between 0.4 and 0.68) when data were analysed crude and nonadjusted for energy content (Table 2). Only the values for protein, cholesterol, and vitamin C were below 0.4. All correlations were significant and the best results were for unsaturated fats (0.68), lipids (0.66), and calcium (0.61). Deattenuation increased all correlation coefficients. A similar effect was verified when values were adjusted for energy content, except for protein, fibre, iron, and zinc.

The agreement of QFFQs and 24hRs was assessed by quartile categorization of adolescents according to energy and nutrient intake. The exact agreement varied from 31.4% (cholesterol) to 47.1% (lipid; Table 3). Extreme disagreement (classification in opposite quartiles) ranged from zero (calcium) to 8.5% (vitamin C). Approximately 70.0% (vitamin C) to 85.7% (calcium) of participants were classified either in the same or in adjacent quartiles. The mean values of exact and exact/adjacent agreement and disagreement were 38.4%, 78.5%, and 4.1%, respectively. Agreements assessed with the weighted* kappa* correction ranged from 0.15 (weak correlation) for vitamin C to 0.45 (moderate) for calcium, with a mean of 0.3.

**Figure 2:**
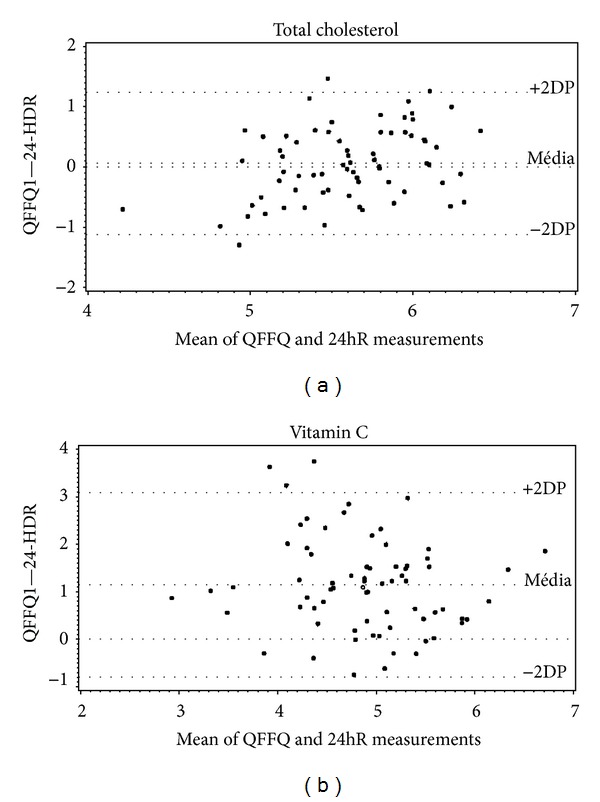
Bland-Altman plots for mean values of total cholesterol and vitamin C intake. These values were obtained from the application of QFFQ and 24hR on type 1 diabetes adolescent patients. Goiânia, Brazil, 2009.

**Figure 3:**
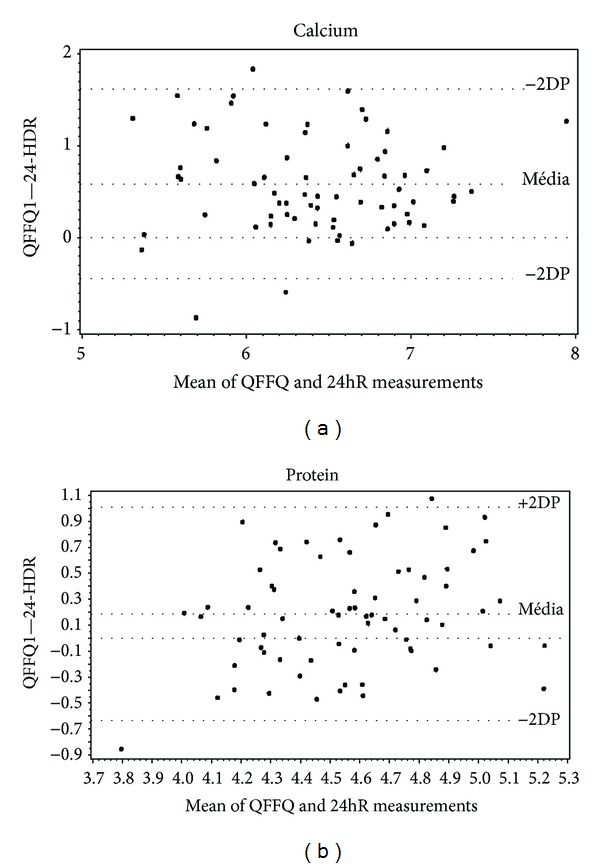
Bland-Altman plots for mean values calcium and protein intake. These values were obtained from the application of QFFQ and 24hR on type 1 diabetes adolescent patients. Goiânia, Brazil, 2009.

Figures 2 and 3 show the Bland-Altman analysis of our measurements, which plots the difference in intake between the two methods against the mean in intake of the two measures for each individual intake. Both axes are logarithmic. The highest validity on visual inspection is that for cholesterol (Figure 2). The major bias is observed for the vitamin C measurements (Figure 2). Validity was acceptable for both protein, which showed the lowest correlation value (0.32), and calcium, which showed the highest (0.75; Figure 3).

**Figure 4:**
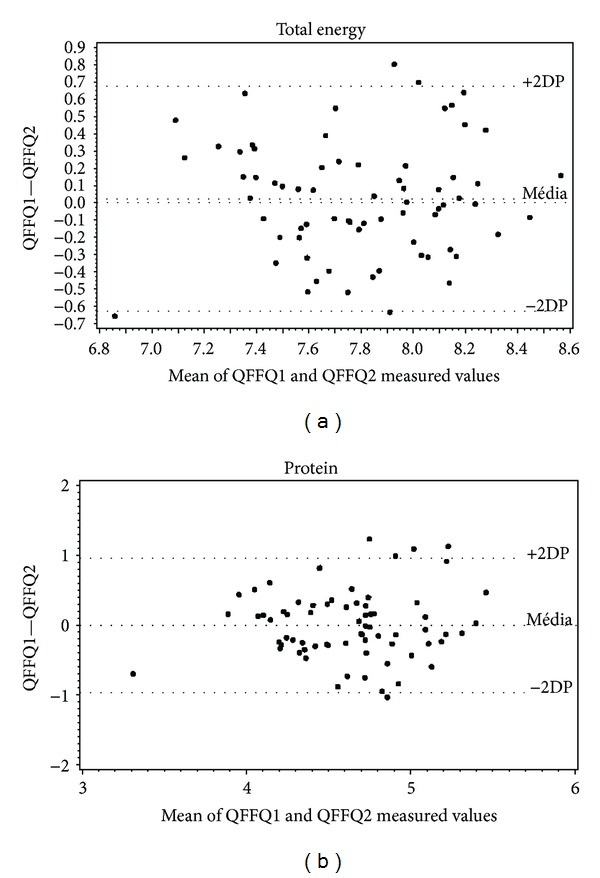
Bland-Altman plots for the mean total energy and protein intake of two QFFQs on type 1 diabetes adolescent patients. Goiânia, Brazil, 2009.

Results for the reproducibility test are presented in Table 4. The ICC ranged from 0.25 (calcium) to 0.65 (lipid), with a mean of 0.46 for nonadjusted values. With the adjustment for energy content, values became lower for lipid, saturated and unsaturated fats, carbohydrate, and protein and higher for cholesterol, fibre, calcium, iron, and zinc. The ICC was not significant for vitamin C. Bland-Altman analysis showed good agreement between both questionnaires of each method for all nutrients (Figure 4).

## 4. Discussion

The applied questionnaire, designed specifically for evaluating the population from our study (Brazilian type 1 diabetic adolescents of a low income population), was able to measure the food intake in the subjects analyzed, with a good reproducibility and high agreement when compared to standard (reference) methods. Adolescents in this study had a* per capita* income lower than half the minimum wage and therefore below the poverty line, whose expenses with health and medications, according to the Brazilian Family Budget Survey (BFBS) for years 2002-3 [19], reach, respectively, 12.45% and 8.78% of familial income. Regarding dietary habits surveys, quantitative frequency of food consumption questionnaires, when compared to reference methods, tends to overestimate food and nutrient intake, especially relative to fruits and thus to vitamin C, as shown in this study [2, 6]. Slater et al. [20], validating a questionnaire for adolescents, observed that intake overestimation may occur when the questionnaire is applied to adolescents. Watson et al. [21], who validated a questionnaire for adolescents in Australia, also reported that the method overestimated energy, macronutrients, and fibres.

Crude Pearson correlation coefficients were similar to those described by Willet [11] (0.5–0.7) to be accepted in validation studies. They were close to those of Watson et al. [21], lower than those of Slater et al. [20], and higher than those of Rodriguez et al. [16] and Kobayashi et al. [22], all of which were carried out in adolescents. Correlation coefficients lower than 0.4 were similar to the results of Rockett et al. [14] for cholesterol, of Riley and Blizzard [23] for protein, and of Rodriguéz et al. [24] for vitamin C. Despite limitations of methods due to their reliance on memory and difficulties in calculating portion size, Hernández-Avila et al. [25] suggest that the observed differences among methods are more likely due to intake frequency rather than portion size.

Energy adjustment corrects nutrient intake for caloric content. When it was applied, the crude correlation coefficients for some nutrients rose, which indicates that energy content is a source for the observed variability. However, the values for protein, zinc, and iron became lower, which can be explained by systematic over- or underestimation of intake.

The intraindividual variation may have influenced results significantly. de Costa et al. [26] noted lower correlation coefficients for protein relative to other macronutrients and energy intake in an adolescent population from Piracicaba, Brazil. They reported that raising the coefficient to 0.9 demanded eleven days of food intake measurements, which is impracticable for validation studies. They nevertheless recommend a minimal of six days for this kind of population.

Epidemiological studies seek to find evidence of association between nutrient intake and the development of chronic diseases. To this end individuals must be classified according to intake levels so that risk factors can be correctly estimated. Therefore, assessing the ability of QFFQ to do this by the agreement between repeated applications of the questionnaire may be more important than correlation analysis. The means for exact agreement and extreme disagreement found in this study were similar to those of Slater et al. [20] for adolescents. The low agreement and high disagreement found for vitamin C are similar to the ones reported by Rodriguéz et al. [24] in adults and by Giacomello et al. [27] in pregnant women with up to seven years of school education.

Rodriguéz et al. [24] contend that 24hRs record dietary intake more precisely. They note that vitamin C is a nutrient difficult to measure due to the high daily variation in the intake of specific items such as fruits and vegetables and to the limited number of applied questionnaires, thus resulting in low correlation coefficients and agreement rates between methods.

Percent agreement values between methods may be a random occurrence. Therefore, alternative measurements are necessary to complement the comparison. The weighted* kappa* is an indicator that corrects agreement values for randomness. In the present study results fell below the recommendation for exact agreement, but all values for extreme disagreement were below 10%. As for the* kappa*, even if vitamin C showed poor agreement scores, the mean value was regular. Voci et al. [15] assessed the validity of a questionnaire by food groups and observed low agreement for fruits and meat. In contrast, Assis et al. [28] found a mean* kappa* of 0.85 for a previous-day questionnaire applied in school-age individuals, but in this case the surveys were carried out at shorter intervals.

Watson et al. [21] found weak to regular agreement in questionnaires retrieved from Australian adolescents which they attributed to the intrinsic limitation of three 24hRs in faithfully registering the intake of items for which there is great within-individual variation. However, Xia et al. [29] showed moderate agreement.

As for the evaluation of agreement by Bland-Altman plots, which are seldom used in QFFQ validation studies, Voci et al. [15], Robinson et al. [30], and the present study found that the questionnaire overestimated values relative to the reference method. Giacomello et al. [27] showed that vitamin C is the nutrient with which methods disagree the most.

ICC values indicate the degree of association and agreement between values from each questionnaire. Our results for this reproducibility assessment were close to those reported by Marchioni et al. [31] and by Robinson et al. [30]. Rodriguez et al. [16] found values higher than 0.5 and Nahas et al. [32] found values higher than 0.8, but in both cases the interval was shorter between consecutive applications of the questionnaire.

It is recommended that the interval between applications should not be too short, lest the individual remembers the answers, or too long, lest there are changes in dietary habits. Our study found results similar to another Brazilian survey that used the same three-month interval [28]. McPherson et al. [33] suggest that the higher the correlation coefficients are, the shorter the interval between applications is, which probably allows for fewer changes in dietary patterns and a higher recall of answers.

Reproducibility studies in adults found higher correlation coefficients. Values between 0.5 and 0.7 are acceptable for such studies, even if they are considered low relative to laboratory studies under controlled conditions [11]. However, children and adolescents are expected to yield lower coefficients, since the difference in nutrient ingestion in adolescent is twice that of adults [20, 24].

Children at seven or eight years of age can report their dietary intake, since by this time they already notice the passage of time and the ingestion of meals. Nevertheless, both older children and adolescents have difficulty in reporting portion size. It is therefore suggested that the ability to quantify ingestion does not depend on age since even adults have problems estimating the food quantities. A complicating factor is that this age range experiences quick changes in feeding habits. It must be considered that studies with adolescents rely on cognitive skills to record intake, but on subjective assessments of portion size [20].

Other reproducibility studies have also reported a reduction of the ICC values when they were adjusted for energy content [20, 31, 34] and this phenomenon is an indicator of systematic error in estimating ingestion, which is common in adolescents [21, 31].

The results plotted on Bland-Altman charts confirm the good agreement between the two QFFQ measurements for each individual. Marchioni and colleagues [31] reported an overestimation bias in the first application, but it was not significant. In the present study, there was a small positive difference for lipids, saturated and unsaturated fats, calcium, and total energy, but fibre, vitamin C, and iron were not underestimated.

Some limitations of our study must be remarked. Due to the sample size it was not possible to divide it according to age, gender, and nutritional status, all of which are factors that can influence intake estimates [31]. To be used on other populations, even on adolescent ones, this QFFQ must be validated again considering socioeconomic characteristics and health status of patients (considering that our population was based on a low income rate, e.g.). Other points considered by the authors as drawbacks are as follows. Although correlation coefficients fell in the “moderate” range, all were significant, especially the values for protein intake. Since the QFFQ significantly overestimated intake relative to the reference method, the obtained results must be analysed cautiously. The greater variability in consumption found in adolescents, which would account for the weaker correlation values, could be minimized by the application of more 24hRs and the use of familiar tools that help respondents to estimate portions and record intake and by motivating patients and helping them to develop skills that facilitate adherence to the survey. Even if perfect agreement is not reached, it must be taken into consideration that, for epidemiological studies, estimates of habitual dietary intake, even if less precise, are more relevant than more accurate measurements of current intake, which fail to capture general exposure trends [34].

## 5. Conclusion

In conclusion, the QFFQ was able to measure usual intake of energy, macronutrients, cholesterol, vitamin C, calcium, iron, and zinc by type 1 diabetes adolescent patients and, as required by epidemiological studies, showed good ability to classify them by intake level. Reproducibility results were also acceptable, confirming that this questionnaire will enable the execution of longitudinal studies that help us to understand clinical outcomes and better to orient and accompany these patients with a view to promoting their healthy growth and development.

## Figures and Tables

**Table 1:** Total energy and nutrient intake means. The means of energy and nutrient intake were estimated from results of two quantitative food frequency questionnaires (QFFQ) and four 24-hour recalls (24hR) filled by adolescents with type 1 diabetes. Goiânia, Brazil, 2009.

Energy and nutrients	QFFQ (*n* = 70)		24hR (*n* = 70)		QFFQ : 24hR ratio	*P* value^†^
	Mean	SD∗	Mean	SD∗		
Total energy						
kJ	10946.22	3673.80	7885.58	2246.09	1.39	0.0001
kcal	2616.21	878.06	1884.70	536.83		
Carbohydrate (g)	312.89	109.94	199.42	63.07	1.57	0.0001
Protein (g)	114.25	45.74	92.39	32.05	1.24	0.0004
Lipid (g)	100.85	40.51	79.71	28.92	1.26	0.0001
Saturated fat (g)	29.84	12.97	23.35	8.79	1.28	0.0001
Unsaturated fat (g)	57.18	22.79	45.77	15.85	1.25	0.0001
Total cholesterol (mg)	327.53	185.21	286.15	127.75	1.14	0.0001
Dietary fibre (g)	51.88	17.37	33.08	13.43	1.57	0.0001
Vitamin C (mg)	314.62	294.94	106.59	86.77	2.95	0.0001
Calcium (mg)	983.28	699.37	542.23	299.52	1.81	0.0001
Iron (mg)	13.30	4.63	9.81	3.71	1.35	0.0001
Zinc (mg)	17.54	8.43	15.36	6.93	1.14	0.0354

∗SD: standard deviation; ^†^Student's *t*-test (significant if *P* < 0.05).

**Table 2:** Pearson correlation coefficients for the comparison between energy and nutrient intake. These values were estimated by QFFQ and 24hR from type 1 diabetes adolescent patients. Goiânia, Brazil, 2009.

Energy and nutrients	Unadjusted				Energy-adjusted			
	Crude	(CI 95%)∗	*Deattenuated* ∗∗	(CI 95%)∗	Crude	(CI 95%)∗	*Deattenuated* ∗∗	(CI 95%)∗
Total energy	0.47	(0.26–0.64)	0.48	(0.28–0.64)	—	—	—	—
Carbohydrate	0.44	(0.22–0.61)	0.45	(0.24–0.62)	0.58	(0.40–0.72)	0.60	(0.43–0.73)
Protein	0.36	(0.14–0.55)	0.38	(0.16–0.56)	0.31	(0.08–0.50)	0.32	(0.09–0.52)
Lipid	0.66	(0.50–0.77)	0.67	(0.51–0.78)	0.74	(0.61–0.83)	0.75	(0.63–0.84)
Saturated fat	0.57	(0.39–0.71)	0.59	(0.41–0.72)	0.58	(0.40–0.72)	0.60	(0.42–0.73)
Unsaturated fat	0.68	(0.53–0.79)	0.69	(0.55–0.80)	0.74	(0.61–0.83)	0.75	(0.62–0.84)
Total cholesterol	0.36	(0.13–0.55)	0.37	(0.15–0.56)	0.38	(0.16–0.56)	0.40	(0.19–0.58)
Dietary fibre	0.56	(0.38–0.70)	0.59	(0.41–0.72)	0.56	(0.37–0.70)	0.58	(0.40–0.72)
Vitamin C	0.33	(0.10–0.52)	0.34	(0.12–0.54)	0.42	(0.20–0.60)	0.44	(0.23–0.61)
Calcium	0.61	(0.44–0.74)	0.62	(0.45–0.75)	0.73	(0.60–0.82)	0.75	(0.62–0.84)
Iron	0.47	(0.27–0.64)	0.49	(0.29–0.65)	0.45	(0.24–0.62)	0.47	(0.26–0.63)
Zinc	0.40	(0.18–0.58)	0.42	(0.20–0.59)	0.38	(0.16–0.57)	0.40	(0.18–0.58)

∗CI = confidence interval; ∗∗intraclass correlation coefficient.

**Table 3:** Agreement measures for energy and nutrient intake. The measures for energy and nutrient intake were estimated by QFFQ and 24hR from type 1 diabetes adolescent patients, categorized into intake quartiles. Results were assessed by the *kappa *test. Goiânia, Brazil, 2009.

Energy and nutrients	Exact agreement (%)	Exact plus adjacent-quartile agreement (%)	Extreme disagreement (%)	Weighted *Kappa *
Total energy	32.8	84.2	5.7	0.29
Carbohydrate	37.1	77.1	5.7	0.27
Protein	32.8	75.7	5.7	0.22
Lipid	47.1	80.0	4.2	0.38
Saturated fat	41.4	84.2	2.8	0.38
Unsaturated fat	45.7	80.0	2.8	0.38
Total cholesterol	31.4	74.2	2.8	0.22
Dietary fibre	37.1	75.7	4.2	0.27
Vitamin C	32.8	70.0	8.5	0.15
Calcium	45.7	85.7	0.0	0.45
Iron	41.4	77.1	4.2	0.31
Zinc	35.7	78.5	2.8	0.29

Mean	38.4	78.5	4.1	0.30

**Table 4:** Reproducibility assessment as measured by the intraclass correlation coefficient (ICC) for energy and nutrient intake. This intraclass correlation coefficient was calculated by the application of two QFFQs per individual on type 1 diabetes adolescent patients. Goiânia, Brazil, 2009.

Energy and nutrients	ICC	CI 95%∗	Adjusted ICC	CI 95%∗
Total energy	0.59	0.416–0.725	—	—
Carbohydrate	0.54	0.348–0.684	0.44	0.234–0.613
Protein	0.33	0.105–0.522	0.29	0.061–0.490
Lipid	0.65	0.493–0.767	0.52	0.330–0.674
Saturated fat	0.56	0.375–0.700	0.41	0.201–0.591
Unsaturated fat	0.65	0.485–0.763	0.56	0.373–0.700
Total cholesterol	0.42	0.211–0.597	0.48	0.280–0.643
Dietary fibre	0.41	0.198–0.588	0.55	0.363–0.694
Vitamin C	0.38	0.166–0.566	0.21	−0.030–0.419
Calcium	0.25	0.014–0.453	0.35	0.125–0.538
Iron	0.38	0.165–0.566	0.41	0.192–0.585
Zinc	0.40	0.184–0.578	0.46	0.254–0.626

Mean	0.46	—	0.43	—

∗CI: confidence interval.

## Abbreviations

24hR: 24-hour recall
BFBS: Brazilian family budget survey
CF: Consumption frequency
DM1: Type 1 diabetes
EOC-CH: Endocrinology outpatient clinic, Clinical Hospital of the Federal University of Goiás
F: Consumption frequency categories
FICF: Free and informed consent formulary
GFU: Goiás Federal University
GGH: Goiânia General Hospital
ICC: Intraclass correlation coefficient
*n*_*x*_: Number of surveys per subject
PC: Percentage contribution
QFFQ: Quantitative food frequency questionnaire
*r*_*d*_: Deattenuated coefficient
*r*_*p*_: Observed coefficient
SAS: Statistical Analysis System
*Sn*: Weights
SPSS: Statistical Package of Social Sciences
*λ*_*x*_: Ratio of intrapersonal variation.
